# Supplementary material for: The fate of early perichondrial cells in developing bones
Source: Nat Commun. 2022 Nov 28;13:7319. doi: 10.1038/s41467-022-34804-6 (PMC9705540; doi:10.1038/s41467-022-34804-6)
Supplement: Supplementary file 1 — Supplementary Information [file 41467_2022_34804_MOESM1_ESM.pdf]

Supplementary Information

The fate of early perichondrial cells in developing bones

Matsushita et al.

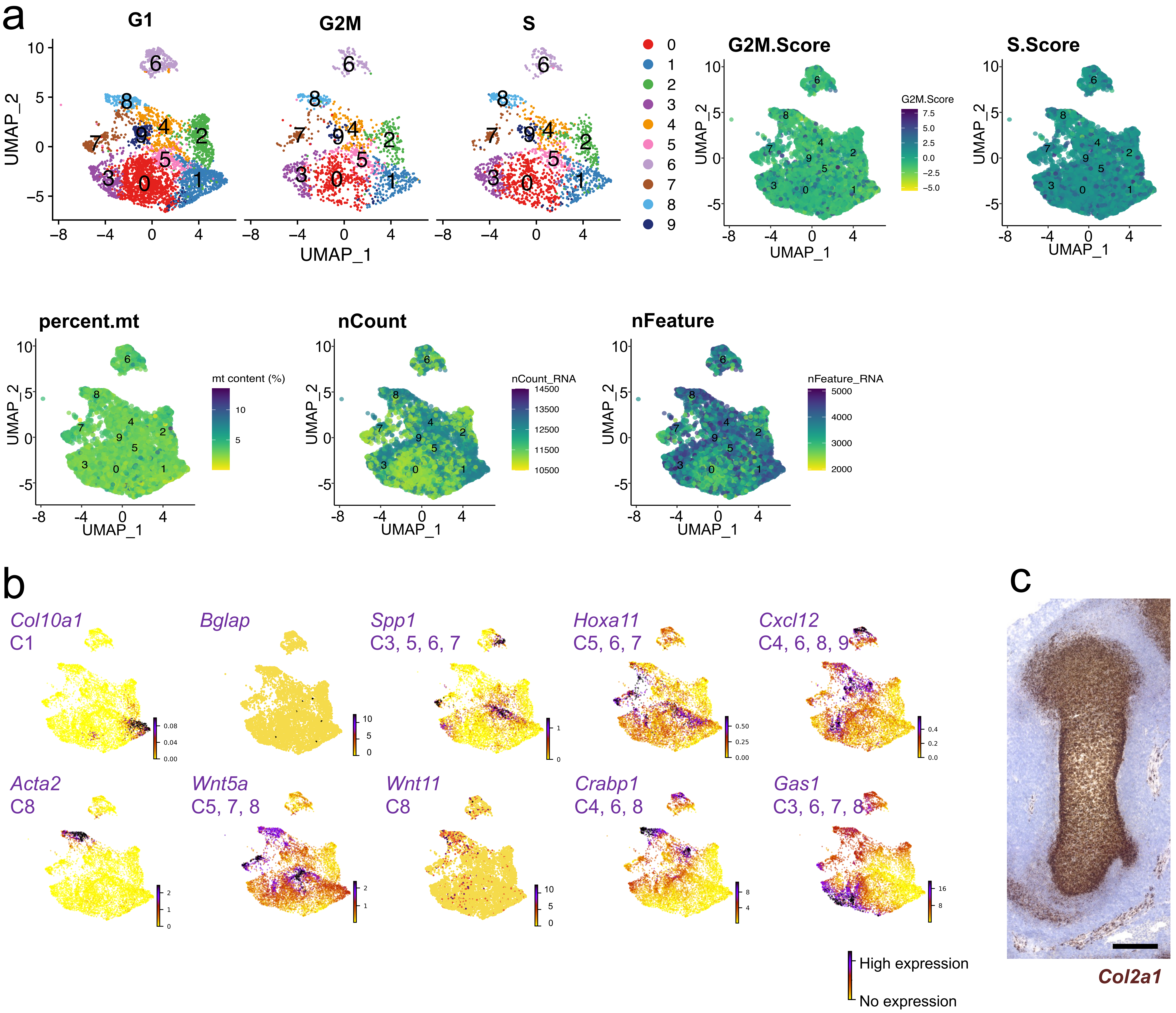

Supplementary Figure 1. Single-cell RNA-seq identifies a perichondrial cluster as a putative cell origin in the fetal cartilage

(a): Top panel: Distribution of cells classified in G1, G2/M, and S phase (left). Feature plots of quantitative score at G2/M and S phase of the cell cycle (right). Bottom panel: color indicates the percentage of mitochondrial content (left), the total number of molecules detected (center), and the number of genes detected (right) in each cell.

(b): Feature plots of representative genes (*Col10a1, Bglap, Spp1, Hoxa11, Cxcl12, Acta2, Wnt5a, Wnt11, Crabp1* and *Gas1*) enriched in each cluster. Violet: high expression, yellow: low expression.

(c): RNAScope analyses of *Col2a1*. Scale bar: 200µm. *n*=4 mice.

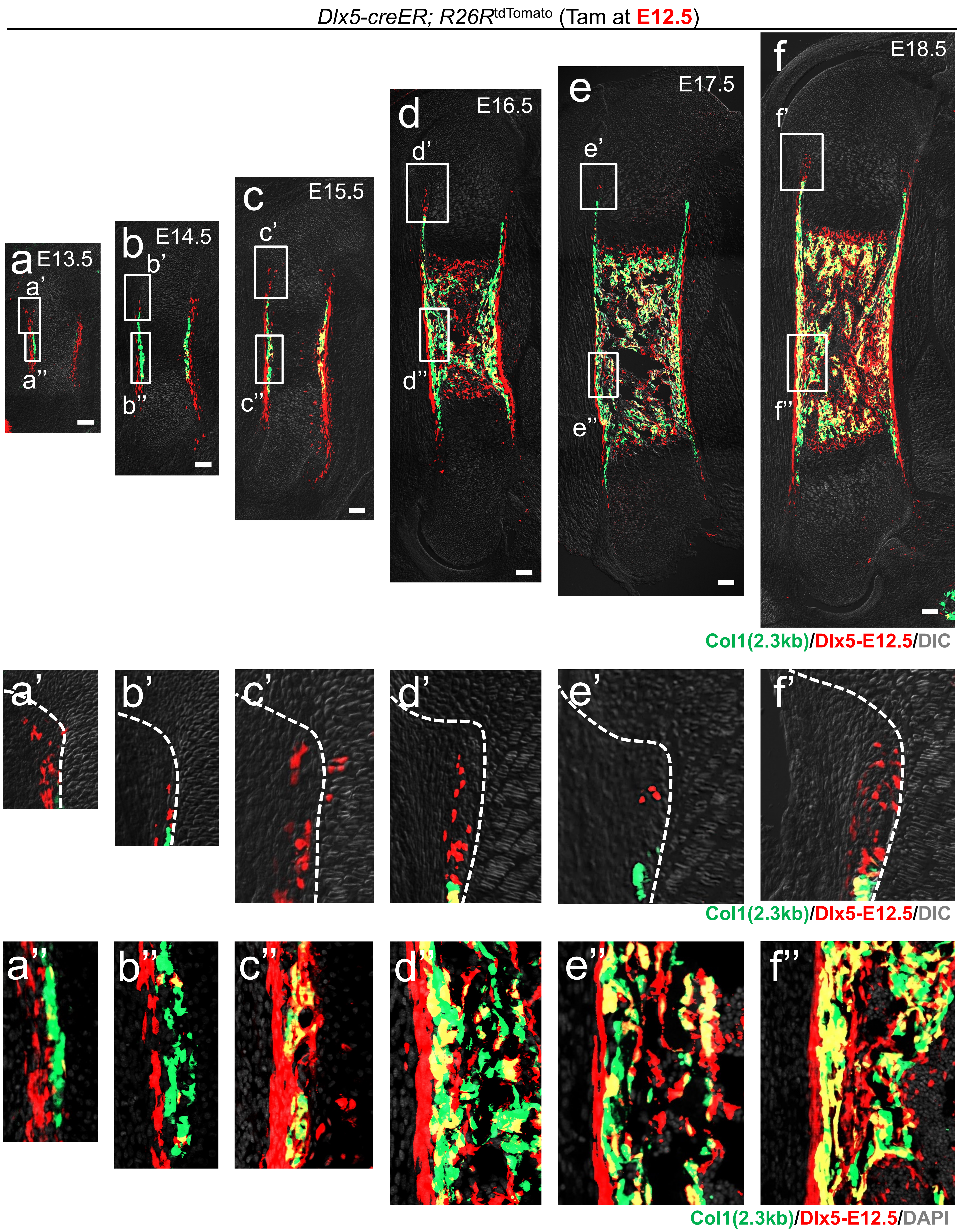

Supplementary Figure 2. Dlx5-creER+ early perichondrial cells contribute to both cortical and marrow stromal compartment

(a-f) Lineage tracing analyses of *Col1a1(2.3kb)-GFP*; *Dlx5-creER; R26R*^{tdTomato} femur (pulsed at E12.5). Migratory path of Dlx5^{CE}-E12.5 cells from the perichondrium to the marrow space by analyzing serial time points from E13.5 to E18.5. E13.5 (a). E14.5 (b), E15.5 (c), E16.5 (d), E17.5 (e) and E18.5 (f). Scale bar: 200µm. *n*=4 mice per each group.

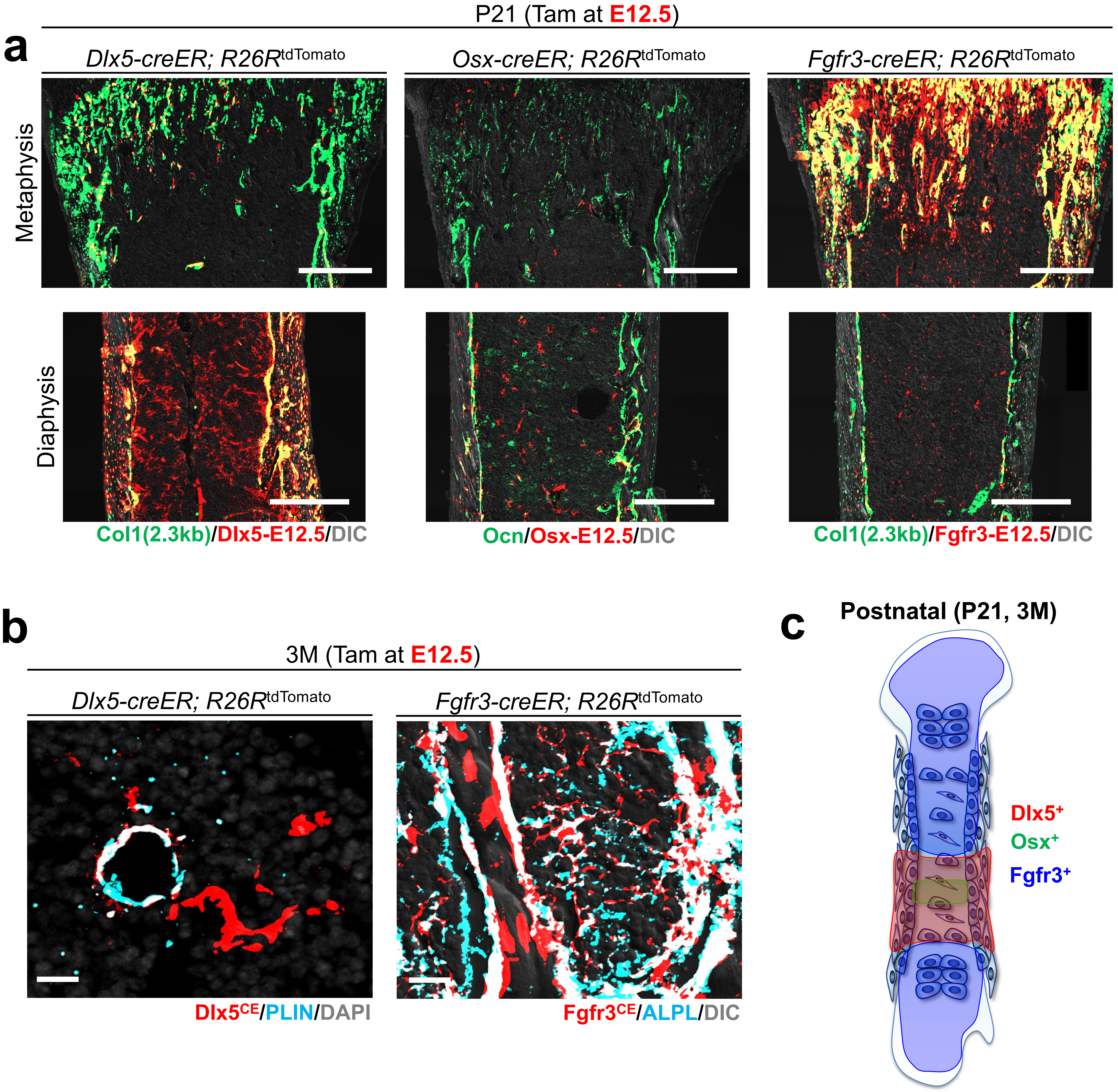

Supplementary Figure 3. Dlx5-creER+ perichondrial cells contribute postnatally to diaphyseal bone marrow stroma

(a) Lineage contribution of fetal (E12.5) Dlx5-creER+, Osx-creER+ or Fgfr3-creER+ cells to osteoblasts and osteocytes at P21. Upper panels: metaphysis. Lower panels: diaphysis. Scale bar: 500µm. *n*=4 mice per each group.
(b) Lineage contribution of fetal (E12.5) Dlx5-creER+ cells to adipocytes and Fgfr3-creER+ cells to osteoblasts at 3M. Immunostaining for PLIN (Dlx5-creER+ cells) and ALPL (Fgfr3-creER+ cells). Scale bar: 20µm. *n*=4 mice per each group.
(c) Diagram depicting lineage contribution of Dlx5-creER+, Osx-creER+ and Fgfr3-creER+ cells in endochondral bone formation, at postnatal stages (P21 and 3M).

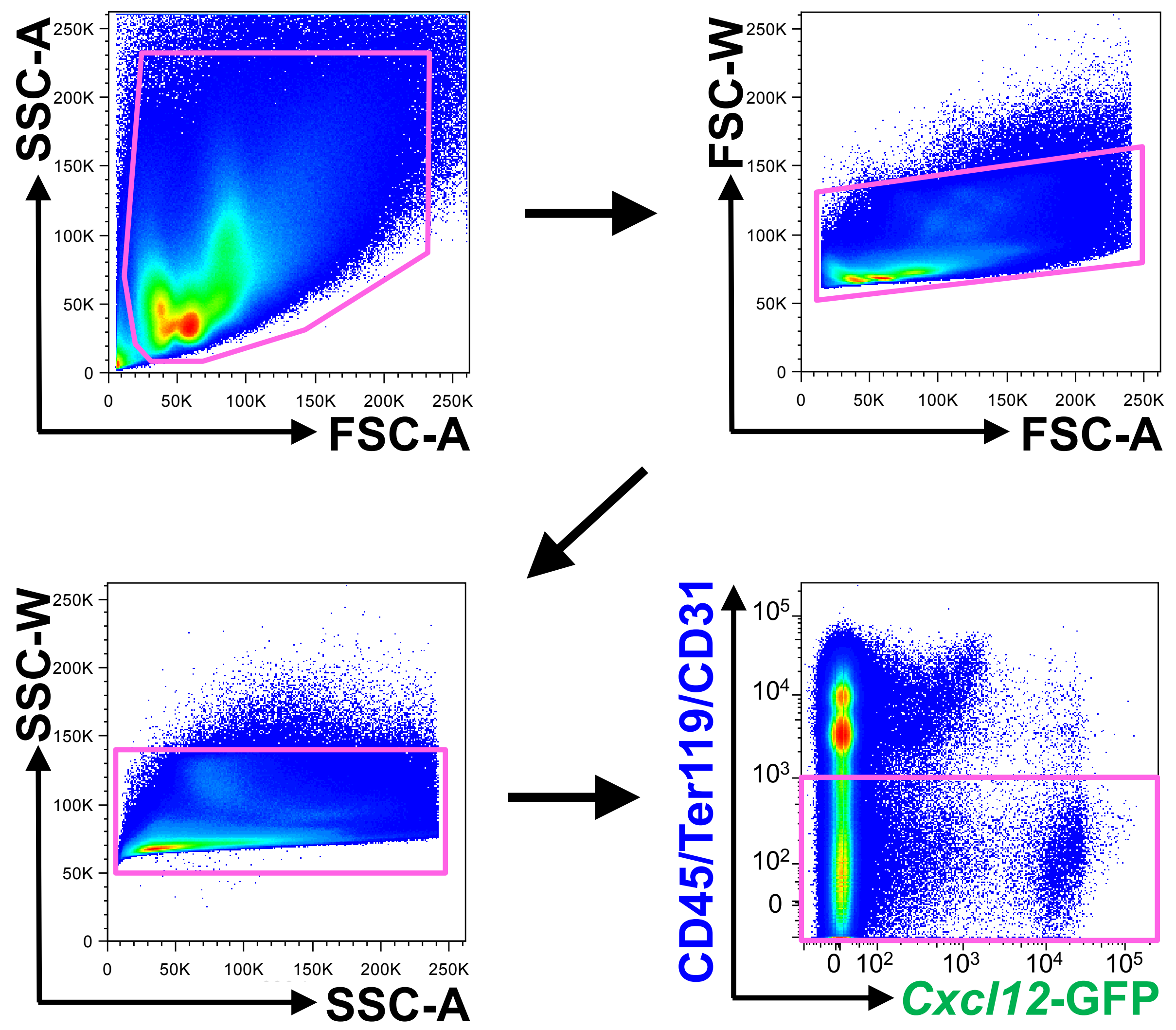

Supplementary Figure 4. Flow cytometry analysis of lineage-marked Cxcl12-GFP^{+} bone marrow stromal cells

Flow cytometry analysis of CD45/Ter119/ CD31^{neg} cells at P21. Gating strategy for Cxcl12-GFP^{+} bone marrow stromal cells isolated from *Cxcl12*$^{GFP/+}$; *Dlx5-creER*; *R26R*^{tdTomato}, *Cxcl12*$^{GFP/+}$; *Osx-creER*; *R26R*^{tdTomato} and *Cxcl12*$^{GFP/+}$; *Fgfr3-creER*; *R26R*^{tdTomato} femurs (pulsed at E12.5).

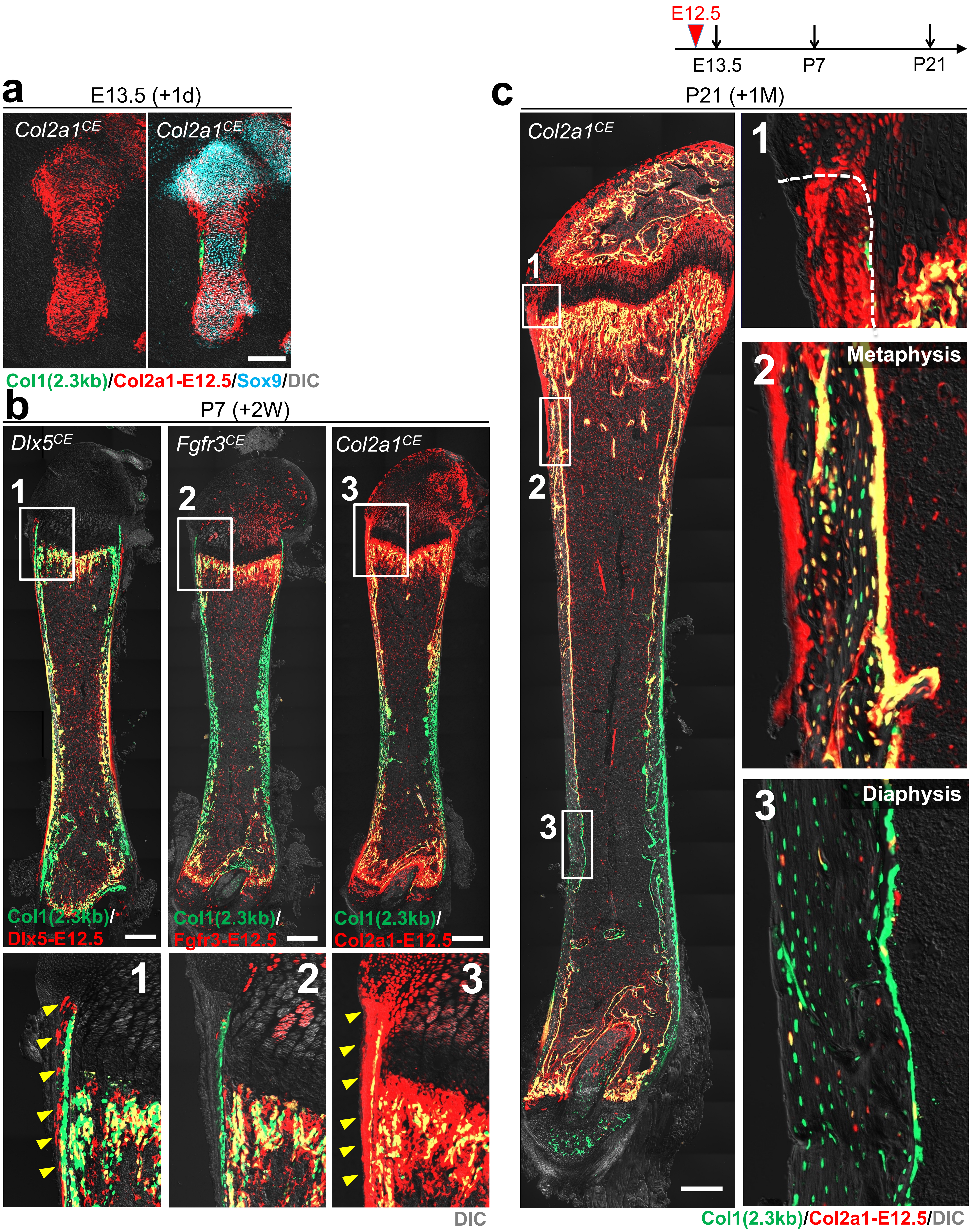

Supplementary Figure 5. Dlx5-creER⁺ perichondrial cells contribute postnatally to diaphyseal bone marrow stroma

(a-c) Cell-fate analysis of Col2a1-creER⁺ cells, pulsed at E12.5.
(a): Cartilage template of *Col1a1(2.3kb)-GFP*; *Col2a1creER*; *R26R*^{tdTomato} femur at E13.5 stained for Sox9. Scale bar: 200µm. *n*=4 mice.
(b): *Col1a1(2.3kb)-GFP*; *Dlx5-creER*; *R26R*^{tdTomato}, *Col1a1(2.3kb)-GFP*; *Fgfr3-creER*; *R26R*^{tdTomato} or *Col1a1(2.3kb)-GFP*; *Col2a1-creER*; *R26R*^{tdTomato} femurs at P7. Lower panels: magnified images of the rectangles. Arrowheads: perichondrial or periosteal cells.
Upper left: *Col1a1(2.3kb)-GFP*; *Col2a1creER*; *R26R*^{tdTomato} femurs at E13.5. Scale bar: 500µm. *n*=4 mice per each group.
(c): *Col1a1(2.3kb)-GFP*; *Col2a1-creER*; *R26R*^{tdTomato} femur at P21. Right panels: magnified views of the rectangles. Dotted line: border of perichondrium and growth plate. Scale bar: 500µm. *n*=4 mice.
