# Supplementary material for: The fate of early perichondrial cells in developing bones
Source: Nat Commun. 2022 Nov 28;13:7319. doi: 10.1038/s41467-022-34804-6 (PMC9705540; doi:10.1038/s41467-022-34804-6)
Supplement: Supplementary file 3 — Reporting Summary [file 41467_2022_34804_MOESM3_ESM.pdf]

Corresponding author(s): Noriaki Ono
Last updated by author(s): Nov 5, 2022

Reporting Summary

Nature Portfolio wishes to improve the reproducibility of the work that we publish. This form provides structure for consistency and transparency in reporting. For further information on Nature Portfolio policies, see our Editorial Policies and the Editorial Policy Checklist.

Statistics

For all statistical analyses, confirm that the following items are present in the figure legend, table legend, main text, or Methods section.

n/a	Confirmed	
☐	☒	The exact sample size (*n*) for each experimental group/condition, given as a discrete number and unit of measurement
☐	☒	A statement on whether measurements were taken from distinct samples or whether the same sample was measured repeatedly
☐	☒	The statistical test(s) used AND whether they are one- or two-sided<br>*Only common tests should be described solely by name; describe more complex techniques in the Methods section.*
☐	☒	A description of all covariates tested
☐	☒	A description of any assumptions or corrections, such as tests of normality and adjustment for multiple comparisons
☐	☒	A full description of the statistical parameters including central tendency (e.g. means) or other basic estimates (e.g. regression coefficient) AND variation (e.g. standard deviation) or associated estimates of uncertainty (e.g. confidence intervals)
☐	☒	For null hypothesis testing, the test statistic (e.g. *F*, *t*, *r*) with confidence intervals, effect sizes, degrees of freedom and *P* value noted<br>*Give P values as exact values whenever suitable.*
☒	☐	For Bayesian analysis, information on the choice of priors and Markov chain Monte Carlo settings
☒	☐	For hierarchical and complex designs, identification of the appropriate level for tests and full reporting of outcomes
☒	☐	Estimates of effect sizes (e.g. Cohen's *d*, Pearson's *r*), indicating how they were calculated

Our web collection on statistics for biologists contains articles on many of the points above.

Software and code

Policy information about availability of computer code

Data collection	ZEN 2 (blue edition, ZEISS), FACSDiva (BD), Cell Ranger 2.2 (10X Genomics)
Data analysis	ZEN 2 (blue edition, ZEISS), Image J 13.0.6 (NIH), FlowJo 9.9.6 (TreeStar), GraphPad Prism 8.4.3 (GraphPad Software), Seurat 3.0, CellChat, velocyto, scVelo, CellRank, DESeq2, EdgeR

For manuscripts utilizing custom algorithms or software that are central to the research but not yet described in published literature, software must be made available to editors and reviewers. We strongly encourage code deposition in a community repository (e.g. GitHub). See the Nature Portfolio guidelines for submitting code & software for further information.

Data

Policy information about availability of data

All manuscripts must include a data availability statement. This statement should provide the following information, where applicable:

- Accession codes, unique identifiers, or web links for publicly available datasets
- A description of any restrictions on data availability
- For clinical datasets or third party data, please ensure that the statement adheres to our policy

The data generated in this study are provided in the Source Data file. The raw data generated during and/or analyzed during the current study are available from the corresponding author on reasonable request. The single cell and bulk RNA-seq data presented herein have been deposited in the National Center for Biotechnology Information (NCBI)'s Gene Expression Omnibus (GEO), and are accessible through GEO Series accession numbers GSE126966 [https://

www.ncbi.nlm.nih.gov/geo/query/acc.cgi?acc=GSE126966], GSE197933 [https://www.ncbi.nlm.nih.gov/geo/query/acc.cgi?acc=GSE197933]. Source data are provided with this paper. Reference genome mm10 (Ensembl release 93) for transcript annotation is publicly available on Ensembl, including a gtf file [http://ftp.ensembl.org/pub/release-93/gtf/mus_musculus/Mus_musculus.GRCm38.93.gtf.gz] and a fasta file [http://ftp.ensembl.org/pub/release-93/fasta/mus_musculus/dna/Mus_musculus.GRCm38.dna_sm.primary_assembly.fa.gz]. The scRNA-seq data presented in this study is accessible from the following link through the cellxgene platform: https://welchlab.dcmb.med.umich.edu/view/Dlx5/col2e.h5ad/. Source data are provided with this paper.

Human research participants

Policy information about studies involving human research participants and Sex and Gender in Research.

Reporting on sex and gender	N/A
Population characteristics	N/A
Recruitment	N/A
Ethics oversight	N/A

Note that full information on the approval of the study protocol must also be provided in the manuscript.

Field-specific reporting

Please select the one below that is the best fit for your research. If you are not sure, read the appropriate sections before making your selection.

☒ Life sciences ☐ Behavioural & social sciences ☐ Ecological, evolutionary & environmental sciences

For a reference copy of the document with all sections, see nature.com/documents/nr-reporting-summary-flat.pdf

Life sciences study design

All studies must disclose on these points even when the disclosure is negative.

Sample size	No statistical method was used to predetermine sample size. We chose the numbers of mice based on the similar research in the field (Nat Commun 11:332, 2020). These sample size give good standard errors of the mean and good statistics to make it unlikely that we miss a biologically important difference between groups.
Data exclusions	Some of the data were excluded from the study because of the pre-established criteria such as problems or failures in identifying correct genotypes or birth dates, and issues unrelated to the intervention of the study such as spontaneous malnutrition. In any case, we consistently used littermate controls with corresponding genotypes in analysis.
Replication	For all data presented in the manuscript, we examined at least three independent biological samples (three different mice) to ensure the reproducibility. For each series of the experiments, all attempts at replication were successful.
Randomization	The experiments were not randomized. We used all the available mice of the desired genotypes. Mice were allocated to particular groups based on results of PCR-genotyping typically performed around one week after birth. Covariates were controlled by considering multiple factors, such as genotypes and general phenotypical data (i.e. body weight). On principle, we did not observe any particular difference among groups.
Blinding	The investigators were not blinded to allocation during experiments and outcome assessment because it was impossible due to following reasons: samples were allocated to particular groups before experiments were initiated based on genotyping results, and given unique identifiers highlighting groups throughout experiments i.e. housing in cages, tissue collections, sample preparation and data acquisition. However, we did not pay particular attention to groups when we were measuring and counting.

Reporting for specific materials, systems and methods

We require information from authors about some types of materials, experimental systems and methods used in many studies. Here, indicate whether each material, system or method listed is relevant to your study. If you are not sure if a list item applies to your research, read the appropriate section before selecting a response.

Materials & experimental systems

n/a	Involved in the study
☐	☒ Antibodies
☒	☐ Eukaryotic cell lines
☒	☐ Palaeontology and archaeology
☐	☒ Animals and other organisms
☒	☐ Clinical data
☒	☐ Dual use research of concern

Methods

n/a	Involved in the study
☒	☐ ChIP-seq
☐	☒ Flow cytometry
☒	☐ MRI-based neuroimaging

Antibodies

Antibodies used

ThermoFisher/eBioscience
eFlour450-conjugated CD31 (390, Cat# 48-0311-82, Lot# 4301770)
eFlour450-conjugated CD45 (30F-11, Cat# 48-0451-82, Lot # 4295770)
eFlour450-conjugated Ter119 (TER-119, Cat# 48-5921-82, Lot# 4295840)
Allophycocyanin (APC)-conjugated CD31 (390, Cat# 17-0311-82, Lot# 4330203)
Allophycocyanin (APC)-conjugated CD45 (30F-11, Cat# 17-0451-82, Lot # 1966484)
Allophycocyanin (APC)-conjugated Ter119 (TER-119, Cat# 17-5921-82, Lot# 4295921)

ThermoFisher/Invitrogen
Alexa Fluor 647 donkey anti-rabbit IgG (H+L) (Cat# A31573, Lot# 1322326)
Alexa Fluor 633 goat anti-rat IgG (H+L) (Cat# A21049, Lot# 679064)

Sigma
Rabbit anti-perilipinA/B polyclonal antibody (Cat#1873, Lot# 018M4869V)

R & D systems
Goat anti ALPL polyclonal antibody (Cat# AF2910, Lot# WYM0116081)

EMD-Millipore
Rabbit anti-Sox9 polyclonal antibody (Cat# AB5535, Lot# 2922429)

Santa Cruz Biotechnology
Rat anti-endomucin (Emcn) monoclonal antibody (V.7C7Cat# sc65495, Lot# C2816)

Abcam
Rabbit anti-Myh3 polyclonal antibody (Cat# ab124205, Lot# GR3250681-1)

Validation

All antibodies used here are commercially available. More detailed information about these antibodies is available on these manufacturers' websites. Briefly, Antibodies purchased from eBioscience were tested by flow cytometry analysis of mouse splenocytes and/or bone marrow cells. Antibodies from Invitrogen, Sigma. R&D systems, EMD-Millipore, Santa Cruz and Abcam were tested by immunohistochemistry.

Animals and other research organisms

Policy information about studies involving animals; ARRIVE guidelines recommended for reporting animal research, and Sex and Gender in Research

Laboratory animals

We used genetically modified mice (mus musculus) for this study. Most of the mouse line have been backcrossed to C57BL/6 background. We used female breeder mice in a FVB/N or C57BL/6 background. Mice with both sexes were used throughout their lifespan (up to 2 years of age). Mouse strains used in the study were as following: Col2a1-cre (JAX003554), Col2a1-creER (JAX006774), Fgfr3-creER (JAX025809), Dlx5-creER (JAX010705), Rosa26-CAG-loxP-stop-loxP-tdTomato (Ai14: R26R-tdTomato, JAX007914), Col1a1(2.3kb)-GFP (JAX013134), Osteocalcin-GFP (JAX017469), Osterix-mCherry (JAX024850), Ptch1-floxed (JAX030494), FVB/NJ (JAX001800) and C57BL/6J (JAX000664) mice were acquired from the Jackson laboratory. Fgfr3-GFP (MMRRC:031901-UCD) mice were acquired from the Mutant Mouse Resource and Research Centers. Osx-creER mice were provided from Dr. Henry Kronenberg. Cxcl12GFP/+ mice were provided from Dr. Takashi Nagasawa. Animal rooms were climate controlled to provide temperatures of 22-23°C, 40-65% of humidity on a 12 h light/dark cycle.

Wild animals

No wild animals were used in the study.

Reporting on sex

Sex was not considered in study design, as the study was initiated and completed before the mice reach sexual maturity. Assigning sex was impossible because the mice were pulsed and analyzed during prenatal periods. Data has not been disaggregated for sex. The number provided in the Reporting Summary includes both male and female mice. For the mice analyzed during postnatal periods, information regarding sex has been collected; however, the data from both sexes were aggregated because they were analyzed before sexual maturity.

Field-collected samples

No field collected samples were used in the study.

Ethics oversight	All procedures were conducted in compliance with the Guidelines for the Care and Use of Laboratory Animals approved by the University of Texas Health Science Center at Houston's Animal Welfare Committee (AWC), protocol AWC-21-0070, and the University of Michigan's Institutional Animal Care and Use Committee (IACUC), protocol 9496.

Note that full information on the approval of the study protocol must also be provided in the manuscript.

Flow Cytometry

Plots

Confirm that:

☒ The axis labels state the marker and fluorochrome used (e.g. CD4-FITC).

☒ The axis scales are clearly visible. Include numbers along axes only for bottom left plot of group (a 'group' is an analysis of identical markers).

☒ All plots are contour plots with outliers or pseudocolor plots.

☒ A numerical value for number of cells or percentage (with statistics) is provided.

Methodology

Sample preparation	Soft tissues and epiphyses were carefully removed from dissected femurs. After removing distal epiphyseal growth plates and cutting off proximal ends, femurs were cut roughly and incubated with 2 Wunsch units of Liberase TM and 1mg of Pronase (Sigma/Roche 10165921001) in 2ml Ca2+, Mg2+-free HBSS at 37oC for 60 min on a shaking incubator (ThermomixerR, Eppendorf). After cell dissociation, cells were mechanically triturated using an 18-gauge needle with a 1ml Luer-Lok syringe (BD) and a pestle with a mortar (Coors Tek), and subsequently filtered through a 70µm cell strainer (BD) into a 50ml tube on ice to prepare single cell suspension. These steps were repeated for 5 times, and dissociated cells were collected in the same tube. Cells were pelleted and resuspended in an appropriate medium for subsequent purposes.
Instrument	BD LSR Fortessa (BDBiosciences)
Software	FACSDiva v8.0.1 (BD) & FlowJo 9.9.6 (TreeStar) software
Cell population abundance	Post-sort purity was not determined
Gating strategy	Single cells were first gated using FSC and SSC denominators. Negative 'unstained' control samples were always used as a reference to determine the demarcation between the positive and negative populations.

☒ Tick this box to confirm that a figure exemplifying the gating strategy is provided in the Supplementary Information.
